# scSAMAC: saliency-adjusted masking induced attention contrastive learning for single-cell clustering

**DOI:** 10.1093/bib/bbaf128

**Published:** 2025-03-25

**Authors:** Bo Li, Yongkang Zhao, Jing Hu, Shihua Zhang, Xiaolong Zhang

**Affiliations:** School of Computer Science and Technology, Wuhan University of Science and Technology, Huangjiahu west road 2#, Wuhan 430065, China; Hubei Province Key Laboratory of Intelligent Information Processing and Real-time Industrial System, Wuhan University of Science and Technology, Huangjiahu west road 2#, Wuhan 430065, China; School of Computer Science and Technology, Wuhan University of Science and Technology, Huangjiahu west road 2#, Wuhan 430065, China; Hubei Province Key Laboratory of Intelligent Information Processing and Real-time Industrial System, Wuhan University of Science and Technology, Huangjiahu west road 2#, Wuhan 430065, China; School of Computer Science and Technology, Wuhan University of Science and Technology, Huangjiahu west road 2#, Wuhan 430065, China; Hubei Province Key Laboratory of Intelligent Information Processing and Real-time Industrial System, Wuhan University of Science and Technology, Huangjiahu west road 2#, Wuhan 430065, China; College of Computer Science, South-Central Minzu University, 182# Minyuan road, Hongshan District, Wuhan 430074, China; School of Computer Science and Technology, Wuhan University of Science and Technology, Huangjiahu west road 2#, Wuhan 430065, China; Hubei Province Key Laboratory of Intelligent Information Processing and Real-time Industrial System, Wuhan University of Science and Technology, Huangjiahu west road 2#, Wuhan 430065, China

**Keywords:** clustering, features, contrastive learning, single cells

## Abstract

Single-cell sequencing technology has enabled researchers to study cellular heterogeneity at the cell level. To facilitate the downstream analysis, clustering single-cell data into subgroups is essential. However, the high dimensionality, sparsity, and dropout events of the data make the clustering challenging. Currently, many deep learning methods have been proposed. Nevertheless, they either fail to fully utilize pairwise distances information between similar cells, or do not adequately capture their feature correlations. They cannot also effectively handle high-dimensional sparse data. Therefore, they are not suitable for high-fidelity clustering, leading to difficulties in analyzing the clear cell types required for downstream analysis. The proposed scSAMAC method integrates contrastive learning and negative binomial losses into a variational autoencoder, extracting features via contrastive unit similarity while preserving the intrinsic characteristics. This enhances the robustness and generalization during the clustering. In the contrastive learning, it constructs a mask module by adopting a negative sample generation method with gene feature saliency adjustment, which selects features more influential in the clustering phase and simulates data missing events. Additionally, it develops a novel loss, which consists of a soft k-means loss, a Wasserstein distance, and a contrastive loss. This fully utilizes data information and improves clustering performance. Furthermore, a multi-head attention mechanism module is applied to the latent variables at each layer of autoencoder to enhance feature correlation, integration, and information repair. Experimental results demonstrate that scSAMAC outperforms several state-of-the-art clustering methods.

## Introduction

Transcriptional activity of genes can explain the unique identities and biological functions of individual cells. Traditional bulk gene expression methods analyze the average transcription levels of bulk cells, ignoring the heterogeneity of individual cells. The rapid development of single-cell sequencing technology has narrowed this gap. Single-cell sequencing provides transcriptomic profiles of individual cells, which are crucial for identifying cell types [1], studying complex biological systems [2], and investigating complex diseases [3].

Clustering, as an unsupervised learning method that measures similarity using different distance metrics, is the fundamental approach for scRNA-seq data analysis. However, the high dimensionality, sparsity [4], and noise events of scRNA-seq data complicate the process of single-cell clustering. Therefore, the development of high-precision cell clustering models is essential. In the past decade, numerous scRNA-seq clustering methods have been proposed. For example, SIMLR [5] clusters cells using spectral clustering based on similarities between samples. Principal component analysis [6] performs dimensionality reduction on the original high-dimensional data to cluster them. Seurat [7] employs the Louvain algorithm to identify clusters on shared nearest-neighbor graphs. ScHFC [8] is a hybrid fuzzy model optimized through natural computing, combining fuzzy C-means and Gath–Geva to enhance clustering performance.

While these methods have indeed improved the performance of single-cell clustering, their limitations have become increasingly evident with the advancement of time. Their clustering performance no longer meets the higher standards demanded by the rapid advancements in single-cell sequencing technology. A higher standard entails more stringent requirements across various clustering evaluation metrics to improve scalability of methods. For single cells, noise and sparsity are common challenges, necessitating modern clustering methods that exhibit robustness to these data characteristics [9]. For instance, some algorithms first perform dimensionality reduction on the raw data, which may lead to the loss of important information. Additionally, when faced with thousands of cells and genes, these methods often require high computational burden, greatly restricting the scalability of single-cell clustering algorithms, thereby affecting downstream analysis.

With the advancement of deep learning, the clustering methods on scRNA-seq data have also diversified. Autoencoder is a classical deep neural network model, which designs an encoder to compress the high-dimensional inputs into low-dimensional representations in a latent subspace, and then reconstructs them by a decoder [10]. To extract more efficient features from the latent subspace, many extended versions of autoencoder have been developed, such as denoising autoencoder (DAE) [11] and variational autoencoder (VAE) [12]. Autoencoder is capable of learning discriminant features from the original inputs, which has also been exploited for scRNA-seq data clustering. Tian *et al*. [13] proposed a DAE-based clustering framework, named scDeepCluster, which maps the count matrix of scRNA-seq data with a nonlinear function into the low-dimensional representations by a zero-inflated negative binomial distribution [14] (ZINB)-based autoencoder, and then performs clustering tasks on the potential space using Kullback–Leibler (KL) divergence.

However, these autoencoder-based algorithms solely focus on investigating the data itself, overlooking the inter-cellular relationships, such as pairwise distances between cells. This leads to lower learning efficiency and poorer clustering results. On the other hand, scziDesk [15] addresses the limitations of models like scDeepCluster by further employing a weighted soft k-means clustering algorithm in the latent space, which forces similar cell types to converge, resulting in superior clustering outcomes. Nonetheless, scziDesk has limitations in handling high-dimensional sparse data and does not improve feature correlation learning for sparse data.

To avoid the problem, a novel approach, termed contrast_sc [16], which considers cell relationships, is presented;it extracts features by contrastively learning the similarity between cells. It first implements representation training and then performs clustering on the embeddings in the second stage. The enhanced representations for each sample are achieved by applying Gaussian noise to the original input data. However, this method still suffers from a classic problem: it overly focuses on the similarity and dissimilarity between samples when learning features, potentially leading to the loss of intrinsic data characteristics. Moreover, randomly adding noise may not be beneficial for extracting important features using the network. In addition, scDGDC [17] employs triplet contrastive learning to dual graph convolutional networks, achieving excellent clustering performance. NsDCC [18] proposes a dual-level contrastive learning method for clustering with good results.

Besides, some methods enhance clustering performance through feature augmentation. For example, scDFC [19] proposes both a structure-based and an attention-guided feature learning modules, both of which improve the performance of clustering. scDeepFc [20] uses a DAE network and a deep graph convolution network to embed high-dimensional gene attribute information and high-order cell–cell topological relation into different low-dimensional representations, and then fuses them to generate a more comprehensive and accurate consensus representation via a deep fusion network. scMFC [21] develops random walk techniques and a cross-view information aggregation mechanism to better capture higher order relationships of the cell graph.

**Figure 1:**
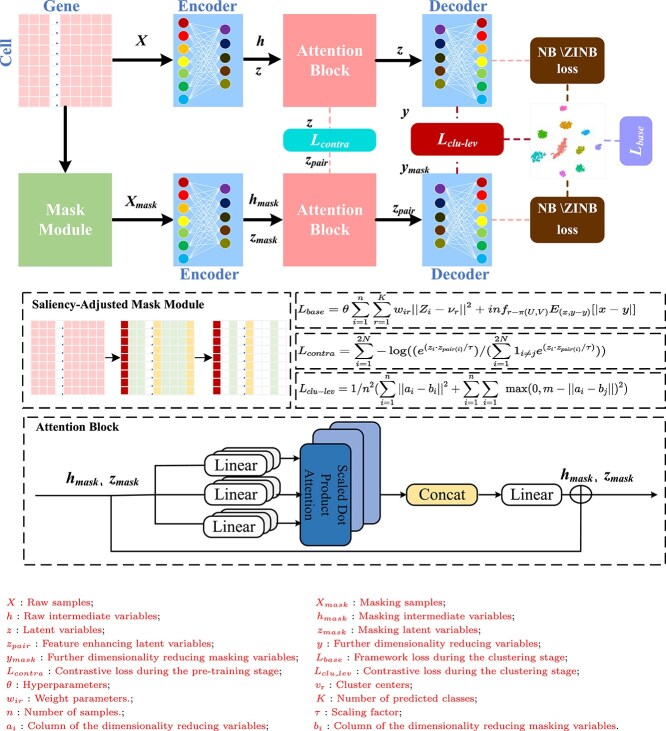
The framework of scSAMAC.

To address the limitations of existing methods, inspired by the aforementioned works, we propose a novel deep framework scSAMAC, shown in Fig. 1, for clustering scRNA-seq data. scSAMAC leverages a saliency-adjusted masking mechanism to generate negative samples for contrastive learning, aiming to capture more distinctive features from scRNA-seq data. Additionally, an attention block is introduced to enhance the intermediate latent features, thereby improving downstream clustering performance. Finally, a novel clustering loss incorporating newly proposed loss functions is employed to further refine clustering outcomes. This framework can resolve the problems related to dropout events and high dimensionality. Meanwhile, it improves the robustness and generalization performance in single-cell clustering. The main contributions of this paper are summarized in three aspects:

1. We construct a mask module, where different masking rates are set for genes based on their expression differences, generating negative samples for contrastive learning. These negative samples are more suitable for VAE framework optimized by negative binomial (NB) loss, preserving significant features for clustering while simulating missing events. Consequently, it enhances clustering performance, robustness, and generalization.
2. We develop a cluster-level loss. By integrating Wasserstein distance and soft k-means loss into it, a hybrid loss is modeled to replace the traditional KL divergence to optimize clustering performance. The cluster-level loss can identify representative features of clusters by leveraging the idea that labels represent the probability of belonging to a specific cluster. Soft k-means loss effectively utilize the pairwise distances between cells. Wasserstein distance is more suitable for sparse data compared with the traditional KL divergence.
3. A block with multi-head attention mechanism is constructed and applied to the latent variables. It extracts feature correlations while retaining some of the original data’s characteristics, preventing excessive feature information loss, which could degrade clustering results. Additionally, the attention block can well utilize existing features to fill in missing data, enabling information integration and repair, thus enhancing data completeness and usability.

## Method

scSAMAC builds a VAE-based deep framework for scRNA-seq data clustering. It consists of a masking module to generate negative samples, two encoders to learn intermediate latent features from the original and masking data, two attention blocks to enhance intermediate latent features via contrastive technique in the pretraining phase, and two contrastive decoders for the final clustering. To optimize the proposed deep network, a contrastive loss is first developed for training, and then a cluster-level loss, combining Wasserstein distance and soft k-means loss, is modeled for clustering. These losses, together with NB or ZINB loss, are used, respectively, in training and clustering to optimize model performance.

scSAMAC is divided into two stages: pretraining and clustering. The original input $ X $ and the masked input $ X_{{mask}} $ are separately passed through the encoder, and the resulting latent variables are then fed into an attention block. The latent variable $ z $, generated from the original input, is then passed to the decoder to compute NB or ZINB loss. In the pretraining phase, the generated $ z $ and $ z_{{pair}} $ are used for contrastive learning to produce the contrast loss. In the clustering phase, instead, $ z $ initially generates $ L_{{base}} $. Subsequently, $ z $ and $ z_{{pair}} $ are each passed through a decoder to match the number of predicted clusters. The resulting $ y $ and $ y_{{mask}} $ are used for column-wise comparison to produce the cluster-level contrast loss.

### Preprocessing of scRNA-seq data

The preprocessing of raw scRNA-seq read count data is also conducted using the Python library SCANPY [22]. Initially, genes lacking expression in any cell are filtered out. Subsequently, size factors are computed to normalize read counts based on library size, ensuring uniform total counts across cells. Formally, denoting the library size (total read counts) of cell $i$ as $s_{i}$, the size factor of cell $i$ is calculated as $s_{i} / \text{median}(s)$. The final step involves logarithmic transformation and scaling of the read counts, facilitating the attainment of unit variance and zero mean in count values. This preprocessed read count matrix serves as the input for our DAE.

### Denoising autoencoder

NB or ZINB is one of the approximation to distribution of scRNA-seq data. They have been applied to extract denoising embeddings and overcome the discrete property and over-dispersion of scRNA-seq dataset: 


(1) \begin{align*} & {{P}_{NB}}({{X}_{n\times m}}|,)=\frac{\Gamma ({{X}_{n\times m}}+\theta )}{\Gamma ({{X}_{n\times m}}+1)\Gamma (\theta )}\times{{\left(\frac{\theta} {\theta +\mu }\right)}^{\theta }}\times{{\left(\frac{\mu }{\theta +\mu }\right)}^{{{X}_{ij}}}}, \end{align*}

(2) \begin{align*} & \scriptsize{P_ {ZINB} ( X_ { n \times m } \ | \pi, \mu, \theta ) = \pi \delta_{ 0 } ( X_ { n \times m } ) + ( 1 - \pi ) \times P_ { N B } ( X_ { n \times m } \ | \mu, \theta ),} \end{align*}

where $X$ denotes the unprocessed raw input data. The mean $\mu $ and dispersion $\theta $ are parameters of distribution, and $\pi $ represents the probability of dropout events in the original counts. The encoder takes $X$ as input, with its output layer generating a reduced-dimensional latent embedding $z$. Conversely, the decoder sub-network takes $z$ as input and produces reconstructions of the original input $X$. Both the encoder and decoder are neural networks based on multi-layer perceptron. Subsequently, three distinct fully connected layers are utilized to fit the mean $\mu $, dispersion $\theta $, and the dropout $\pi $: 


(3) \begin{align*} & Z={{W}_{encoder}}(X+\epsilon), \end{align*}

(4) \begin{align*} & D={{W}_{decoder}}(Z), \end{align*}

(5) \begin{align*} & \mu =F_{diag}\left[ exp^{(D{W}_{\mu})} \right], \end{align*}

(6) \begin{align*} & \theta =exp^{(D{{W}_{\theta }})}, \end{align*}

(7) \begin{align*} & {\pi = F_{sigmoid} ( D W_ { \pi } ),} \end{align*}

where $\epsilon $ represents the random Gaussian noise (0 mean and 0.01 variance), $F_{diag}$ refers to the diagonal matrix construction, and $F_{sigmoid}$ is the sigmoid function. Three different fully connected layers are nonlinear activated by diverse ways. The mean $\mu $ and dispersion $\theta $ are nonnegative, hence exponential function is chosen as an activation function. In order to prevent overfitting, the mean $\mu $ is normalized to the same extent with the raw count size for each cell. The dropout probability $\pi $ should be in a reasonable scale (0–1), so a sigmoid is used for standardization. All the $W$ represent the learnable parameters. Finally, the loss function of DAE is the negative log of NB or ZINB likelihood: 


(8) \begin{align*} & {{L}_{nb}}\left( \mu,\theta \mid X \right)=\log ({{P}_{NB}}({{X}_{n\times m}}\ |\mu,\theta )), \end{align*}

(9) \begin{align*} & {{L_ { z i n b }} ( \pi, \mu, \theta | X ) = \log ( P_ { Z IN B } ( X_ { n \times m } | \pi, \mu, \theta ) ).} \end{align*}

### Saliency-adjusted masking module

In contrastive learning, selecting effective negative samples is essential for enhancing model performance. We introduce a specialized masking strategy named saliency-adjusted to generate these optimal negative samples. In this approach, the standardized input matrix is processed through a masking module to produce negative samples for contrastive learning. The module begins by calculating the column sums of the matrix, where the column with the highest sum is identified as the “most significant” gene column. Based on these sums, we categorize genes into three types: columns with sums closest to the most significant gene are labeled as “significant” gene columns, those with moderately close sums to it as “moderately significant” gene columns, and those with sums farthest from the most significant gene as “least significant” gene columns. We vary masking probabilities across different gene columns. Highly significant gene columns are masked with the lowest probability, minimizing their chances of being set to zero. Columns of moderate significance have a higher probability of zeroing, while the least significant gene columns are masked with the highest probability. The generated negative samples are incorporated during both the pretraining and clustering stages. This saliency-adjusted masking strategy for the input matrix retains attributes critical for clustering and training while enhancing data heterogeneity. By preserving a diverse range of negative samples, this approach helps the model to generalize effectively to unseen data and to mitigate the risk of over-fitting. Furthermore, it also partially addresses the issue of false zeros introduced by dropout events.

### Attention block

Attention mechanism has shown its effectiveness in natural language processing [23], playing a significant role in extracting features related to sample correlation. Here, we also construct an attention block by combining multi-head self-attention mechanism with residual strategy, which is applied before each latent variable layer of the encoder as follows: 


(10) \begin{align*} & Q=K=V={{H}^{l}}, \end{align*}

(11) \begin{align*} & {{Q}_{i}}^{l}=QW_{i}^{{{Q}^{l}}},{{K}_{i}}^{l}=KW_{i}^{{{K}^{l}}},{{V}_{i}}^{l}=VW_{i}^{{{V}^{l}}}, \end{align*}

where $H^{l}$ represents the input of the $l$th layer of the latent variables. $Q$ is query, $K$ is key, and $V$ is value, with the $l$th layer output ${{Q}_{i}}^{l}$, ${{K}_{i}}^{l}$, and ${{V}_{i}}^{l}$, respectively. $W_{i}^{{{Q}^{l}}}$, $W_{i}^{{{K}^{l}}}$, and $W_{i}^{{{V}^{l}}}$ are the weight matrices for $Q$, $K$, and $V$.

Attention scores are 


(12) \begin{align*}& {{a_{i}}^{l} = F_{softmax} ( \frac{ Q_ { i }^{l} {K_ { i }^{l}}^ { T }} { \sqrt{ d_ { k }} } ) V_ { i }^{l},}\end{align*}

where $a^{l}$ is the output head, $F_{softmax}$ represents the softmax function, and $d_ { k }$ is the scaling factor. 


(13) \begin{align*}& F_{multiHead}({Q^{l}},{K^{l}},{V^{l}}) = F_{concat}( a_ { 1 }^{l},a_ { 2 }^{l}\ldots,{a^{l}}_{M}){W^{l}},\end{align*}

where $W^{l}$ is the weight matrix used to map the concatenated multi-head output to the final dimension, $F_{concat}$ represents the vector concatenation function, and $F_{multiHead}$ refers to the multi-head attention function.

The output should be 


(14) \begin{align*}& {H_ { attention }^{l}}=F_{multiHead}({{Q}^{l}},{{K}^{l}},{{V}^{l}})+{{H}^{l}},\end{align*}

where $M$ denotes the number of heads, and $H_ { attention }^{l}$ represents the output enhanced data handled by the attention block. For those high-dimensional data, there may exist redundant or correlated features. The multi-head attention strategy can effectively capture the correlation between them and improve feature representation learning. This is particularly important for sparse data, where many features may have zero values. Therefore, the multi-head attention helps identify features more important for the target task, while the residual mechanism retains some features of the original data to prevent the deterioration of clustering results due to excessive feature losing.

### Loss function

To effectively utilize the information such as between-cell similarity and to address both issues as the asymmetry in traditional clustering processes and the meaningless KL divergence values for distributions, we employ a novel composite loss function here: 


(15) \begin{align*}& {{L}_{{base}}}=\theta \sum\limits_{i=1}^{n}{\sum\limits_{r=1}^{K}{{{w}_{ir}}}}||{{z}_{i}}-{{\nu }_{r}}|{{|}^{2}}+W_{d}(U,V),\end{align*}

where $z$ represents low-dimensional latent variables, $v_{r}$ denotes the cluster center in latent space, $K$ is the predicted number of clusters, $\theta $ is a trade-off parameter, $W_{d}$ is Wasserstein distance between the real distribution $U$ and the target distribution $V$, and $w_{ir}$ is the weight defined as 


(16) \begin{align*}& {{\tilde{w}}_{ir}}=\frac{e^{(-||{{Z}_{i}}-{{\nu }_{r}}|{{|}^{2}})}}{\sum\limits_{k=1}^{k}{e}^{(-||{{Z}_{i}}-{{\nu }_{k}}|{{|}^{2}})}}.\end{align*}

To expedite the convergence, we also perform weight inflation to it: 


(17) \begin{align*}& {{w}_{ir}}=\frac{\tilde{w}_{ij}^{2}}{\sum\limits_{j=1}^{K}{(\tilde{w}_{ij}^{2})}}.\end{align*}

For Wasserstein distance $W_{d}$, its advantages are

1. Wasserstein distance delineates the minimal cost of moving from one probability density to another by capturing the differences between distribution patterns effectively.
2. For any two nonoverlapping distributions, Wasserstein distance can still balance their differences, while KL divergence may be undefined outside the function.
3. Wasserstein distance possesses symmetry and the triangle inequality, addressing the issues of asymmetry and non-transitivity in KL divergence.

It is expressed as 


(18) \begin{align*}& W_{d}(U,V)=in{{f}_{\gamma \sim\prod{(U,V)}}}{{E}_{(x,y\sim\gamma )}}\left[ |x-y| \right],\end{align*}

where *inf* refers to the largest lower bound, and $\gamma \sim (U, V)$ denotes all possible joint distributions between the distributions $U$ and $V$. For each possible joint distribution $\gamma $, $(x, y) \sim \gamma $ means that $x$ and $y$ are sampled from $\gamma $. $E$ represents the expectation of the sample on the distance $|x-y|$ under the joint distribution $\gamma $.

### Contrastive loss in the pretraining phase

In image processing, contrastive learning is commonly used to learn image representations in a low-dimensional subspace [24], causing similar images to be close, while dissimilar images apart. This learning paradigm can be applied to various related tasks. As a result, we extend contrastive learning to single-cell clustering.

We apply the aforementioned masking strategy to the input matrix to generate negative samples $X_{{mask}}$. These negative samples are then fed into a VAE, and the latent variable $z_{{pair}}$ is obtained from the last layer of the encoder. Similarly, the unprocessed input $X$ undergoes the same process to obtain the latent variable $z$. The contrastive loss is calculated as 


(19) \begin{align*}& {{L}_{contra}}=\sum\limits_{i=1}^{2N}{-\log (({e^{({{z}_{i}}\cdot{{z}_{pair(i)}}/\tau} )} /{{(\sum\limits_{i=1}^{2N}{{{1}_{i\ne j}}}e^{({{z}_{i}}\cdot{{z}_{p\text{ai}r(i)}}/\tau )}}})}).\end{align*}

### Cluster-level contrastive loss

At this stage, we adhere to the principle of “labels as representations.” When projecting data samples into a space with dimensions equal to the number of clusters, the $i$th element of the feature vector can be interpreted as the probability of the sample belonging to the $i$th cluster. Consequently, the feature vector represents the soft labels of the sample.

Therefore, we apply the generated negative samples and positive samples, as described above, through a VAE to obtain the latent variables $z_{{pair}}$ and $z$. These latent variables are then fed into the corresponding decoder, where the number of output features equals the predicted number of clusters obtained after pretraining. Consequently, we obtain $y_{{mask}}$ and $y$, which are used for subsequent clustering.

The clustering loss function is then calculated as follows. For each dimension $i$, we calculate the squared Euclidean distance between the $i$th columns of $y_{{mask}}$ and $y$, and obtain their sum: 


(20) \begin{align*}& {{L}_{pos}}=\sum\limits_{i=1}^{n}{||{{a}_{i}}-{{b}_{i}}|{{|}^{2}}},\end{align*}

where $a_{i}$ and $b_{i}$ are the $i$th columns of $y_{{mask}}$ and $y$, respectively.

For all different column pairs $(i, j)$, we can compute the Euclidean distance between the two columns, using the margin parameter $m$ to adjust the following negative loss: 


(21) \begin{align*}& {{L}_{neg}}=\sum\limits_{i=1}^{n}{\sum\limits_{j\ne i}{\max}} {{(0,-m||{{a}_{i}}-{{b}_{j}}||)}^{2}}.\end{align*}

We then combine the losses for both the positive and negative sample pairs with the following formulation as ${L}_{clu-lev}$: 


(22) \begin{align*}& {{L}_{clu-lev}}=\frac{1}{{{n}^{2}}}\left( {{L}_{pos}}+{{L}_{neg}} \right).\end{align*}

Then, the loss for the entire learning process is comprised of the pretraining loss and the clustering loss, which are stated below: 


(23) \begin{align*} & {{L}_{pret\text{r}ain}}=\mu{{L}_{contra}}+{{L}_{nb}}, \end{align*}

(24) \begin{align*} & {{L}_{cluster}}={{L}_{base}}+{{L}_{nb}}+\omega{{L}_{clu-lev}}. \end{align*}

${L}_{zinb}$ is often adopted to train the AE framework, which is also considered in the proposed method by substituting ${L}_{zinb}$ for ${L}_{nb}$ to make comparisons in the following experiments.

## Experiments and results

### Datasets

In experiments, we used 10 single-cell datasets for clustering, as detailed in Table 1.

**Table 1:** Summary of datasets

Dataset	GSE/ID	Cells	Genes	Categories
Human2	GSM2230758	1724	20 125	14
Mouse_ES_cell	GSE65525	2717	24 046	4
CITE_CBMC	SRP073767	8617	2000	15
Klein	GSE65525	2417	24 175	4
Zeisel	GSE60361	3005	19 972	5
Human3	SRP073767	3605	20 125	14
Human4	GSM2230757	1303	20 125	14
Q_10x_Limb_Muscle	GSE109774	3909	23 341	6
QS_seq2_Trachea	GSE109774	1350	19 992	4
Shekhar_Mouse_Retina	GSE81905	27 499	13 166	19

These datasets cover a wide range of single-cell data types, allowing for a comprehensive evaluation of the clustering performance of the proposed method. It includes both small-scale and large-scale cell counts, data with a higher number of genes, and multi-category data. Especially, a very large dataset with 27 499 cells and 13 166 genes, i.e. Shekhar_Mouse_Retina, is also introduced for experiments. Additionally, these datasets are annotated with ground truth labels to evaluate the clustering performance.

### Evaluation metrics

All clustering results are measured by Adjusted Rand Index (ARI) [25], Normalized Mutual Information (NMI), and Accuracy (CA). Given the two clustering assignments $U$ and $V$ on a set with $n$ data points, which have $C_{U}$ and $C_{V}$ clusters, respectively, NMI is defined as the mutual information between $U$ and $V$ divided by the entropy of the clustering $U$ and $V$. Specifically, 


(25) \begin{align*}& NMI=\frac{\sum\nolimits_{\text{p}=1}^{{{C}_{U}}}{\sum\nolimits_{q=1}^{{{C}_{V}}}{|{{U}_{p}}\cap{{V}_{q}}|}}\log \frac{n|{{U}_{p}}\cap{{V}_{q}}|}{|{{U}_{p}}|\times |{{V}_{q}}|}}{\max (-\sum\limits_{p=1}^{{{C}_{U}}}{|{{U}_{p}}|\log \frac{|{{U}_{p}}|}{n}},-\sum\limits_{q=1}^{{{C}_{V}}}{|{{V}_{q}}|\log \frac{|{{V}_{q}}|}{n}})}.\end{align*}

CA is defined as the best matching between a cluster assignment and the ground truth assignment. Given a data point $i$, let $l_{i}$ be the ground truth label and $u_{i}$ be the assignment of the clustering algorithm; CA is then defined as 


(26) \begin{align*}& CA=\underset{m}{\mathop{\max}}\,\frac{\sum\limits_{i=1}^{n}{1}\left\{ {{l}_{i}}=m({{u}_{i}}) \right\}}{n},\end{align*}

where $n$ is the number of data points, and $m$ ranges over all possible one-to-one mappings between cluster assignments and true labels. The best mapping can be efficiently found using the Hungarian algorithm [26].

Rand Index (RI) [27] is a simple measure of agreement between two cluster assignments $U$ and $V$. ARI corrects it for the lack of a constant value of RI when the cluster assignments are selected randomly [28]. ARI is calculated using four quantities. Specifically, we define:

1. The number of pairs of two objects of the same group in both $U$ and $V$: $a$
2. The number of pairs of two objects of different groups in both $U$ and $V$: $b$
3. The number of pairs of two objects of the same group in $U$ but in different groups in $V$: $c$
4. The number of pairs of two objects of different groups in $U$ but in the same group in $V$: $d$

ARI is formally defined as 


(27) \begin{align*}& ARI=\frac{\left( \begin{matrix} n \\ 2 \\ \end{matrix} \right)(a+d)-[(a+b)(a+c)+(c+d)(b+d)]}{\left( \begin{matrix} n \\ 2 \\ \end{matrix} \right)-[(a+b)(a+c)+(c+d)(b+d)]}.\end{align*}

### Results

**Figure 2:**
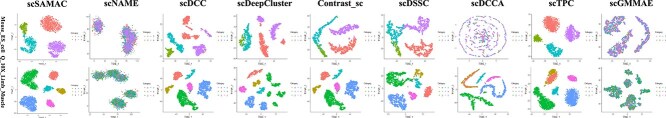
T-SNE visualization results on two datasets.

**Figure 3:**
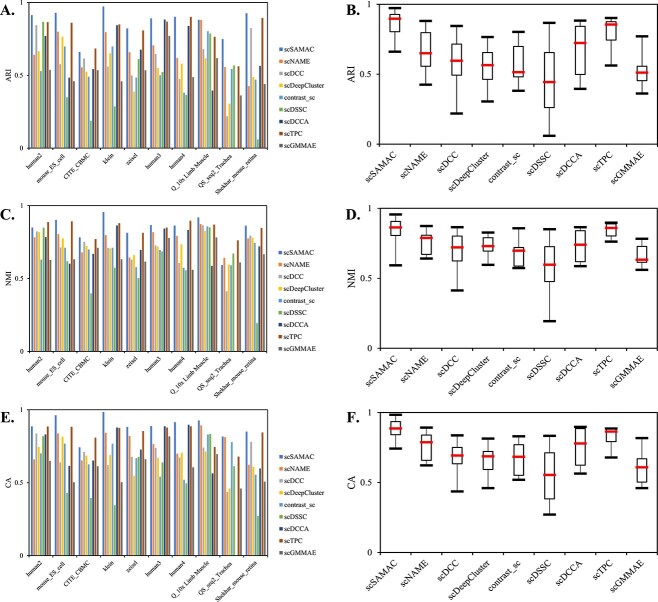
Comparison of clustering results across 10 datasets using 9 different methods. Figures A, C, and E show bar charts of the three evaluation metrics for different methods across various datasets. Figures B, D, and F present box plots of the three evaluation metrics for the different methods.

To justify the effectiveness of our proposed method, we compared it with eight state-of-the-art methods: scNAME [29], scDCC [30], scDeepCluster, contrast_sc, scDSSC [31], scDCCA [32], scTPC [33], and scGMMAE [34]. We also evaluated the results of scSAMAC using NB loss, as well as ZINB loss under the condition that other factors remain unchanged. We used ARI, NMI, and CA to assess their performance, where the higher scores indicate better performance. The performance results across 10 datasets for each method are presented in Tables 2, 3, and 4. The visualizations on two datasets by t-SNE are shown in Fig. 2. Additionally, ARI, NMI, and CA are illustrated in Fig. 3 with bars and boxes, respectively.

**Table 2:** Comparisons of ARI across different methods

Dataset/Method	scSAMAC(NB)	scSAMAC(ZINB)	scNAME	scDCC	scDeepCluster	contrast_sc	scDSSC	scDCCA	scTPC	scGMMAE
human2	**0.9131**	0.8322	0.641	0.8445	0.6658	0.5283	0.8669	0.7704	0.8652	0.5373
mouse_ES_cell	**0.9288**	0.8204	0.7994	0.5769	0.7651	0.6979	0.3491	0.4834	0.8603	0.459
CITE_CBMC	**0.6607**	0.5089	0.5547	0.6152	0.5245	0.4905	0.1876	0.543	0.6838	0.535
klein	**0.9724**	0.8733	0.7963	0.5596	0.6511	0.6979	0.286	0.8436	0.8492	0.459
zeisel	**0.8216**	0.7584	0.6588	0.4991	0.3871	0.4847	0.611	0.6759	0.8078	0.535
human3	**0.8915**	0.8908	0.7063	0.6461	0.5499	0.5005	0.5221	0.883	0.8688	0.7708
human4	**0.902**	0.8324	0.6196	0.4742	0.5791	0.3812	0.3647	0.8385	0.9013	0.4877
Q_10x_Limb_Muscle	**0.8812**	0.8113	0.8802	0.6791	0.6166	0.8025	0.7868	0.3952	0.7645	0.6174
QS_seq2_Trachea	**0.7487**	0.4669	0.5571	0.2192	0.3052	0.5433	0.5673	–	0.5618	0.3619
Shekhar_Mouse_Retina	**0.9265**	0.8322	0.4252	0.8245	0.4888	0.4696	0.0594	0.5648	0.8937	0.4398

**Table 3:** Comparison of NMI across different methods

Dataset/Method	scSAMAC(NB)	scSAMAC(ZINB)	scNAME	scDCC	scDeepCluster	contrast_sc	scDSSC	scDCCA	scTPC	scGMMAE
human2	0.8496	0.7980	0.7832	0.8239	0.8183	0.6296	0.8485	0.7835	**0.8872**	0.627
mouse_ES_cell	**0.9023**	0.8451	0.8047	0.7142	0.7748	0.7117	0.6189	0.6015	0.8931	0.6322
CITE_CBMC	**0.7831**	0.7123	0.6794	0.7519	0.7242	0.6993	0.3976	0.6699	0.7706	0.7107
klein	**0.9557**	0.8907	0.7977	0.7101	0.7064	0.7117	0.574	0.8645	0.8797	0.6322
zeisel	**0.8139**	0.7959	0.6444	0.6296	0.6601	0.578	0.5028	0.6956	0.8138	0.6157
human3	**0.8669**	0.8637	0.8183	0.7273	0.7202	0.6952	0.6853	0.8426	0.8495	0.7782
human4	0.8627	0.8192	0.793	0.6064	0.7352	0.5733	0.5565	0.8333	**0.8969**	0.5601
Q_10x_Limb_Muscle	**0.92**	0.9007	0.8737	0.8646	0.8269	0.8574	0.8506	0.5865	0.8701	0.7821
QS_seq2_Trachea	0.5923	0.5209	0.6414	0.4129	0.5957	0.5908	**0.6708**	–	0.7622	0.6112
Shekhar_Mouse_Retina	0.8632	**0.8644**	0.775	0.7942	0.7805	0.7454	0.1953	0.7205	0.8471	0.6671

**Table 4:** Comparison of CA across different methods

Dataset/Method	scSAMAC(NB)	scSAMAC(ZINB)	scNAME	scDCC	scDeepCluster	contrast_sc	scDSSC	scDCCA	scTPC	scGMMAE
human2	**0.8863**	0.844	0.6607	0.8376	0.7454	0.7007	0.8184	0.8312	0.885	0.6491
mouse_ES_cell	**0.9623**	0.8723	0.8377	0.6386	0.8149	0.7674	0.4299	0.615	0.8833	0.5028
CITE_CBMC	0.7428	0.6146	0.6526	0.7104	0.6853	0.6258	0.3948	0.6517	**0.8083**	0.6131
klein	**0.9849**	0.9006	0.8425	0.6209	0.6894	0.7674	0.3456	0.8785	0.876	0.5028
zeisel	**0.8825**	0.8146	0.82	0.6772	0.5474	0.6682	0.6769	0.7278	0.8539	0.6609
human3	**0.8882**	0.8874	0.7645	0.738	0.6699	0.5417	0.6391	0.8871	0.8786	0.8178
human4	**0.9148**	0.8465	0.6984	0.6724	0.7061	0.5203	0.4958	0.8979	0.8864	0.6055
Q_10x_Limb_Muscle	**0.9268**	0.845	0.8926	0.7406	0.7137	0.8306	0.834	0.5641	0.745	0.6958
QS_seq2_Trachea	**0.8163**	0.6711	0.8122	0.4363	0.46	0.7778	0.6133	–	0.6793	0.46
Shekhar_Mouse_Retina	0.8505	**0.853**	0.6232	0.7802	0.609	0.5545	0.2712	0.5978	0.8446	0.5075

As shown in Tables 2, 3, and 4, it is evident that the proposed method, scSAMAC, generally outperforms the other eight methods across 10 datasets:

- scSAMAC consistently achieves the highest ARI values in nearly all 10 datasets.
- scSAMAC demonstrates comparatively better performance on CA, achieving the highest scores across all methods except for scTPC on CITE_CBMC.
- Although scSAMAC shows a relatively lower NMI value on QS_seq2_Trachea, human2, and human4, it maintains high scores in ARI and CA for these data sets and performs well in NMI on other datasets.

In Fig. 2, the results after visualization demonstrate a better clustering performance of scSAMAC than those of other comparison methods.

In addition, as shown in Fig. 3A, C, and E, scSAMAC is also superior to other comparison methods. Figure 3B, D, and F display that scSAMAC significantly outperforms other clustering methods on certain datasets, particularly showing substantial advantages over all three evaluation metrics on zeisel dataset. Despite exhibiting lower scores in specific metrics for a few datasets, scSAMAC overall shows the best stability in clustering performance. For instance, scSAMAC displays the smallest range between upper and lower limits and the least fluctuation in CA. The median values (indicated by the red lines in the box plots) across the three evaluation criteria further emphasize the superiority of scSAMAC.

As shown in Tables 2, 3, and 4, the clustering results of scSAMAC using NB loss slightly underperform those of scSAMAC with ZINB loss by <1% on two clustering metrics for the Shekhar_Mouse_Retina dataset. However, on the other nine datasets, scSAMAC with NB loss consistently outperforms scSAMAC with ZINB loss across all three evaluation metrics.

### Running time

Moreover, we compare the runtime of the scSAMAC with other methods, all executed on an RTX 4090 GPU (24GB), supported by a 16 vCPU Intel(R) Xeon(R) Platinum 8352V CPU @ 2.10GHz, 120GB of RAM, and running on an Ubuntu 18.04.5 LTS operating system, as shown in Table 5. It reveals that scSAMAC, along with other deep learning-based methods such as scDeepCluster, does not demonstrate a runtime advantage over the contrast_sc algorithm. Moreover, scSAMAC requires more time than both scDeepCluster and scDSSC. The increased runtime in scSAMAC is attributed to the computational demands of the masking strategy and negative sample generation, both of which require the longer processing time. At the same time, scSAMAC with NB loss not only shows advantages in clustering results compared with scSAMAC with ZINB loss, but it is also slightly faster. However, leveraging optimized hardware or alternative strategies could potentially reduce the runtime of deep learning methods, as a promising direction for future improvements.

**Table 5:** Comparison of running time across different methods

Dataset/Method	scSAMAC(NB)	scSAMAC(ZINB)	scNAME	scDCC	scDeepCluster	contrast_sc	scDSSC	scDCCA	scTPC	scGMMAE
human2	258s	264S	59s	305s	221s	1s	78s	523s	241s	7s
mouse_ES_cell	1052s	1140	241s	1121s	874s	1s	175s	1984s	909s	15s
CITE_CBMC	383s	399S	75s	422s	299s	1s	251s	701s	314s	8s
klein	977s	1075S	201s	1005s	785s	1s	611s	1726s	865s	17s
zeisel	724s	792S	145s	823s	661s	1s	593s	1890s	688s	15s
human3	874s	958S	156s	963s	728s	1s	546s	1572s	752s	20s
human4	228s	233S	32s	241s	175s	1s	155s	431s	182s	6s
Q_10x_Limb_Muscle	827s	869s	178s	853s	651s	1s	581s	1582s	709s	17s
QS_seq2_Trachea	645s	671s	121s	771s	562s	1s	514s	1332s	683s	16s
Shekhar_Mouse_Retina	3385s	3641s	723s	3794s	3227s	5s	3098s	7561s	3341s	60s

### Parameters setting

There are some parameters involved in the proposed scSAMAC algorithm. Thus, in the experiments, they are set via parameter sensitivity analysis.

#### VAE module

For the whole VAE, we partially referred to some parameters as scDeepCluster. For instance, we set the training epochs to 400 during the pretraining process, and the learning rate to 1 during the clustering stage. However, to accommodate the contrastive learning loss, we adjusted the learning rate for pretraining to 0.0001.

#### Attention block

In the attention module, we set the number of heads for the multi-head attention mechanism to 8 for both the latent variables with dimensions of 64 and 32, yielding the best results.

#### Masking module

**Figure 4:**
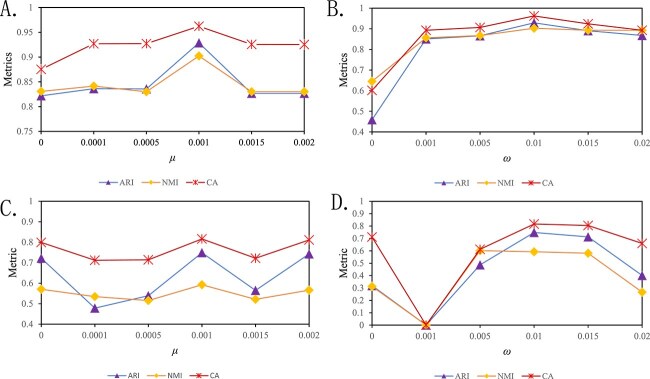
Sensitivity analysis. A. The curves of ARI, NMI, and CA with different $\mu $ on mouse_ES_cell; B. the curves of ARI, NMI, and CA with different $\omega $ on mouse_ES_cell; C. the curves of ARI, NMI, and CA with different $\mu $ on QS_seq2_Trachea; D. the curves of ARI, NMI, and CA with different $\omega $ on QS_seq2_Trachea.

In generating contrastive phase, we adopted a saliency-adjusted masking strategy. Specifically, we labeled the gene columns with the highest column sum as the most salient gene columns. Columns with sums less than one-tenth of the most salient column were labeled as non-salient columns. Columns with sums between one-tenth and three-tenths of the most salient column were labeled as moderately salient columns, and the remaining columns were labeled as salient columns. Subsequently, we zeroed out some columns based on their saliency: 10% probability for salient columns, 30% for moderately salient columns, and 60% for non-salient columns. All these parameters are adjustable. Since we introduced contrastive loss alongside NB loss during the pretraining, we designed a hyper-parameter $\mu $ to balance their relationship. Through a series of parameter sensitivity experiments (refer to Fig. 4A and C), we set it to 0.001. In the clustering phase, we set the hyperparameter $ \omega $ for the cluster-level loss to 0.01 (refer to Fig. 4B and D).

### Convergence

**Figure 5:**
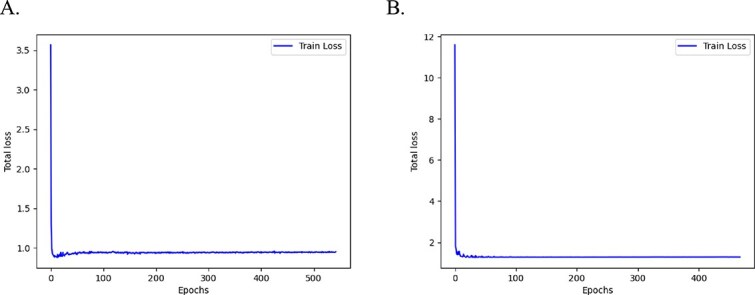
Convergence analysis. A. The loss curve for mouse_ES_cell dataset. B. The loss curve for QS_seq2_Trachea dataset.

To further observe the convergence of the scSAMAC framework, we plotted the total loss during the clustering stage on mouse_ES_cell and QS_seq2_Trachea datasets. As shown in Fig. 5, it can be seen that with the increasing of iterations, the proposed model gradually converges and maintains stability with minor fluctuations.

### Ablation study

**Figure 6:**
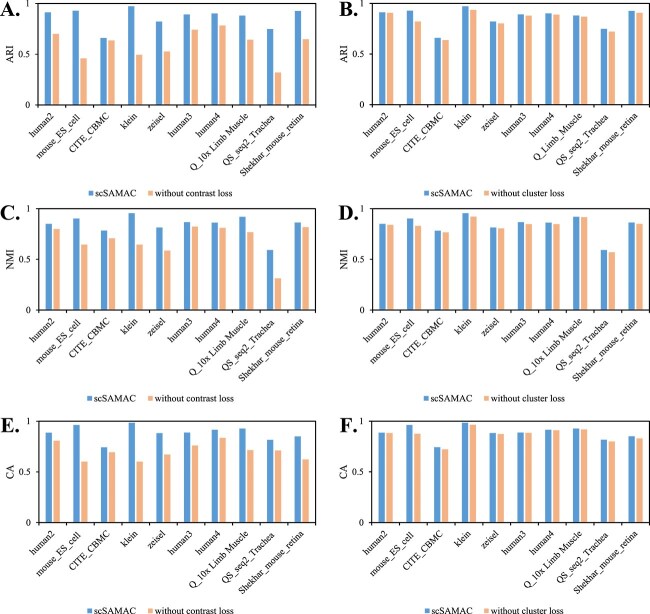
Ablation study. A. ARI results comparison with contrast loss function and without contrast loss function; B. ARI results comparison with cluster-level loss and without cluster-level loss; C. NMI results comparison with contrast loss function and without contrast loss function; D. ARI results comparison with cluster-level loss and without cluster-level loss; E. CA results comparison with contrast function and without contrast loss function; F. CA results comparison with cluster-level loss and without cluster-level loss.

To evaluate the contributions of the newly added modules in scSAMAC and to measure their effectiveness, we conducted ablation experiments on the saliency-adjusted masking module used for contrastive loss and the cluster-level loss module. We compared ARI, NMI, and CA before and after the ablation, as shown in Fig. 6A–E, and F. It is evident that these two modules contribute to improving clustering performance. Methods retaining the new modules demonstrate higher ARI, NMI, and CA values compared with those by removing the corresponding modules. Furthermore, it is clear that the contrastive loss with the new masking module significantly enhances clustering performance.

**Figure 7:**
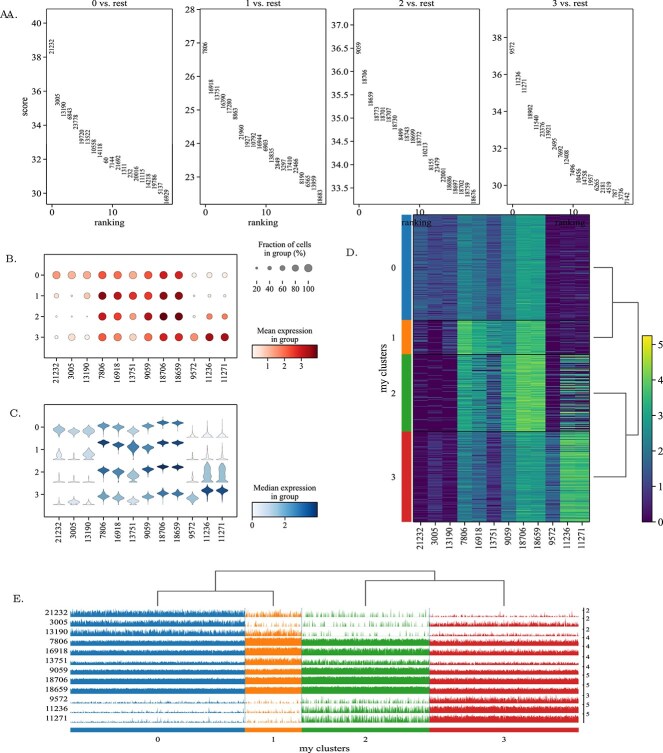
DE analysis of zeisel dataset. In subgraph A, the plot title represents the category index, and the numbers within the plot indicate the sample gene indices. In subgraph B, C, and D, the numbers on the horizontal axis denote gene indices, while the vertical axis shows category indices. In subgraph E, the horizontal axis indicates category indices, and the left vertical axis represents gene indices. The subgraph A is the visualization result of the top 20 marker genes detected by Wilcoxonin in each cell cluster. The subgraph B is a violin diagram, which reflects the distribution of marker genes expression. The subgraph C is a bubble graph, which clearly depicts the proportion of the expression of each marker gene in each cell cluster. The subgraph D is a heat map, which portrays the specific expression of marker genes corresponding to each cell cluster. The subgraph E is clustering trajectory plot.

### Differential expression analysis

Differential expression (DE) is key to analyzing scRNA-seq data. In this section, we adopted klein dataset to evaluate the performance of scSAMAC for DE. Specifically, scSAMAC employed the Wilcoxon test to detect potential marker genes for each cluster, where the top 20 marker genes were selected and shown in Fig. 7A. To visualize the expression levels of the top-ranked marker genes within each cluster, we used several plotting methods: violin plots (Fig. 7B) illustrate the distribution of marker gene expression; bubble plots (Fig. 7C) show the proportion of cells expressing the top three marker genes; and heatmaps (Fig. 7D) depict the expression levels of the top three marker genes across different clusters. These visualizations help to observe the proportion and distribution of marker gene expression in different clusters, providing a comprehensive view of the DE patterns in scRNA-seq data.

From the visualization of marker genes in each cluster, it is evident that each cell cluster has specific marker genes. These marker genes can be used to effectively cluster cells. Notably, the marker genes for each cluster are almost unique, strongly supporting our masking strategy. This uniqueness enhances the possibility of applying targeted masking techniques to preserve significant features during training, which improves the overall clustering performance and robustness of our model.

### Dropout-robust analysis

**Figure 8:**
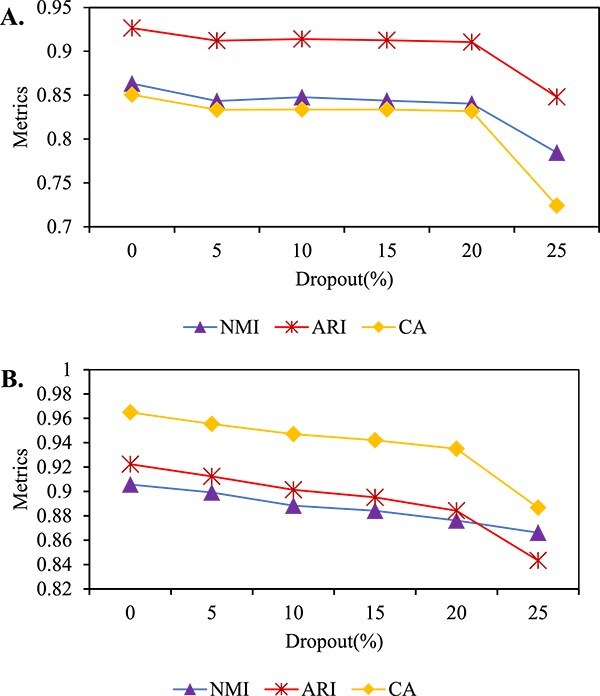
Dropout-robust analysis. A. A curve showing the variation of three evaluation metrics with different dropout rates on Klein dataset. B. A curve showing the variation of three evaluation metrics with different dropout rates on mouse_ES_cell dataset.

To evaluate scSAMAC’s ability to handle dropout events, we selected two standard dataset, klein and mouse_ES_cell, applying masking rates of 0%, 5%, 10%, 15%, 20%, and 25% to simulate the impact of dropout events on them. The clustering results after applying these dropout rates are shown in Fig. 8. It is evident that, for both datasets, scSAMAC’s clustering performance declines with the increasing of dropout rates. However, when the dropout rate remains below 20%, the decline is not significant, indicating that scSAMAC effectively manages low occurring dropout events.

## Discussion and conclusion

To achieve better single-cell clustering performance, this study introduces a novel VAE model for clustering, termed scSAMAC, which employs a new masking method for contrastive learning. In both the pretraining and clustering phases, we use the saliency-adjusted masking module to generate negative samples and conduct contrastive learning during pretraining, combined with NB or ZINB loss. To further optimize clustering performance, we replace the traditional KL divergence with soft k-means loss, the Wasserstein distance, and a newly proposed cluster-level loss in the clustering phase. Additionally, we incorporate an attention module in the latent variable layers of the encoder to well extract features from scRNA-seq data. Experiments on several benchmark datasets with other state-of-the-art algorithms demonstrate the high clustering performance of the proposed algorithm, where ARI, NMI, and CA are used to evaluate clustering effectiveness. Our masking method implements a dynamic masking strategy, adapting to various single-cell data. The introduction of attention blocks enhances data integrity and improves data correlation learning, along with new loss functions, enabling the handling of more complex single-cell data. Thus, we believe that the proposed method will be beneficial for future single-cell clustering applications.

However, scSAMAC model still has some limitations. Firstly, the generation of negative samples involves a high degree of randomness, requiring multiple experiments to achieve ideal results. Additionally, we often observe that the initial clustering results generated after pretraining are quite good, but improvements during the clustering phase are limited or even decline, which demands more loss functions to optimize the clustering phase and enhance clustering results. Furthermore, although scSAMAC demonstrated reasonable effectiveness on single-cell data, it is time-consuming and less stable when compared with methods like SHARP [35] and DUBStepR [36], which excel at fast and efficient clustering in datasets with millions of samples. scSAMAC, as a deep learning method, has disadvantages in computational burden compared with shallow methods like SHARP and DUBStepR, and it faces scalability challenges due to its time-consuming negative sample generation within the masking module of the VAE framework, coupled with the contrastive learning technique. Moreover, the negative sample generation during the masking process may be less stable for large datasets, further limiting scSAMAC’s extensibility. Consequently, further study on deep learning models, including scSAMAC, is necessary to successfully leverage scRNA-seq data to advance human health.

**Key Points**

- A deep framework is constructed for single-cell sequence data clustering, where a VAE module, a masking module, and an attention block contrastive learning module are contained.
- In the mask module, different masking rates are set for genes based on their expression differences, generating negative samples for contrastive learning.
- An attention block contrastive learning module is applied to the latent variables, by which the feature correlations can be extracted while retaining some of the original data’s characteristics and preventing excessive feature losing that could degrade clustering results.
- We replace the traditional KL divergence with the Wasserstein distance, a soft k-means loss, and a cluster-level contrastive loss function for clustering.

## Data Availability

All source code used in our experiments has been deposited at https://github.com/AmateurAntCode/scSAMAC-cluster.git. The scRNA-seq datasets that support the findings of this study are available at https://www.ncbi.nlm.nih.gov/.
